# Supplementary material for: Interactions of N- and C-terminal parts of Ana1 permitting centriole duplication but not elongation
Source: bioRxiv. 2024 Oct 31:2024.10.28.620588. Preprint. [Version 1] doi: 10.1101/2024.10.28.620588 (PMC11565839; doi:10.1101/2024.10.28.620588)

bioRxiv preprint doi: https://doi.org/10.1101/2024.10.28.620588; this version posted October 31, 2024. The copyright holder for this preprint (which was not certified by peer review) is the author/funder, who has granted bioRxiv a license to display the preprint in perpetuity. It is made available under aCC-BY 4.0 International license.

Figure 1- figure supplement 1

(A) Semiquantitative reverse transcription PCR shows transcription of the *ana1* gene in wild type (*WT), ana1*^{null}, *ana1*$^{\Delta 1\text{-}762}$, *ana1*^{mecB} flies. Primers were specific to sequences encoding the N-terminal part and C-terminal part of Ana1. Total mRNA was extracted from freshly eclosed adult males for each genotype. cDNA library was created by reverse transcription. Equal concentrations of cDNA-s were added to a PCR reaction performed under non saturating conditions. Primers specific to *rpL17* were used for loading control. MWM: molecular weight marker; kb: kilobase.

(B) Coordination assay for individual flies. The walking abilities of n=10 individual flies of each genotype were observed under microscope without anaesthesia. Flies were raised and tested at 25 ^{o}C. Means±SD and *p* values of two-tailed, unpaired t-tests are shown for significant differences are shown. For *WT vs ana1*^{null} and *ana1*$^{\Delta 1\text{-}762}$ *p* value can't be calculated, as values in both columns are identical.

bioRxiv preprint doi: https://doi.org/10.1101/2024.10.28.620588; this version posted October 31, 2024. The copyright holder for this preprint (which was not certified by peer review) is the author/funder, who has granted bioRxiv a license to display the preprint in perpetuity. It is made available under aCC-BY 4.0 International license.

Figure 2- figure supplement 1

(A) Climbing assays of indicated transgenic flies expressing Ana1 fragments in an *ana1*^{null} background. Cohorts of 10 flies were scored for their ability to climb 5 cm in 30 s. Means±SD are shown for n=10 flies were investigated in N=2 independent experiments. *P* values indicated for significant differences. Flies were raised and tested at 25 ^{o}C.

Figure 3- figure supplement 1

Overview of chordotonal organs in wild type (*WT*), *ana1*^{null}, *ana1*^{mecB} and *ana1*^{mecB}*; ana1*$^{715-1729}$ femoral chordotonal organs imaged by electron microscopy.

Figure 4- figure supplement 1

bioRxiv preprint doi: https://doi.org/10.1101/2024.10.28.620588; this version posted October 31, 2024. The copyright holder for this preprint (which was not certified by peer review) is the author/funder, who has granted bioRxiv a license to display the preprint in perpetuity. It is made available under aCC-BY 4.0 International license.

(A) Fertiliy of *WT* and *ana1*$^{1-935}$*; ana1*$^{715-1729}$ males tested by scoring the progeny of individual flies. Males were individually mated with *WT* females over 5 days at 25 ^{o}C. Means±SD are shown for n=10 flies per genotype, the *P* value of two tailed, unpaired t tests is indicated for significant differences.

(B) Quantification of centriole lengths in *endo-ana1 (Ctrl)*, *ana1*$^{1-935}$ and *ana1*$^{1-935}$*; ana1*$^{715-1729}$ round spermatids using tAna1-RFP as marker. n=45 centrioles were measured from N=3 testes. Means±SD are shown, *P* values of two tailed, unpaired t tests are shown for significant differences.

(C) Quantification of centriole structures marked by Ana1-RFP or Ana1$^{1-935}$-RFP in *endo-ana1 (Ctrl)*, *ana1*$^{1-935}$ and *ana1*$^{1-935}$*; ana1*$^{715-1729}$ mature primary spermatocyte cysts. N=3-6 cysts were scored. Means±SD are shown, *P* values of two tailed, unpaired t tests are shown for significant difference.

(D) Representative primary spermatocytes in meiosis 1 from *endo-ana1 (Ctrl)*, *ana1*$^{1-935}$ and *ana1*$^{1-935}$*; ana1*$^{715-1729}$ flies. n=3 meiotic cyts per genotype were examined from different testes with similar results. Centrososmes are marked by Ana1-RFP or Ana1$^{1-935}$-RFP (red) and Asl (green) and γ-Tubulin, clone GTU88 (white). Scale bar, 10 µm.

(E) Complementation tests scoring centriole length. Centrioles marked by Ana1-RFP (red) and Asl (green) were observed in primary mature spermatocytes. n=3 testes was examined per genotype with similar results.

Figure 4- figure supplement 2

(A) Representative mature primary spermatocyte cysts expressing Sas6-GFP (green) together with full length Ana1-RFP (red) or the Ana1$^{1-935}$-RFP fragment (red) in *ana1 an null* background.

(B) Magnified examples of the centriole structures marked with Sas6 (green) and Ana1 or Ana1 1-935 fragment (red)

(C) Quantification of centriole structures marked by both Ana1-RFP or Ana1$^{1-935}$-RFP and Sas6-GFP in *endo-ana1 (Ctrl)*, *ana1*$^{1-935}$ mature primary spermatocyte cysts. n=3 cysts were scored from N=2 testes. Means±SD are shown, P values of two tailed, unpaired t tests are shown for significant differences.

bioRxiv preprint doi: https://doi.org/10.1101/2024.10.28.620588; this version posted October 31, 2024. The copyright holder for this preprint (which was not certified by peer review) is the author/funder, who has granted bioRxiv a license to display the preprint in perpetuity. It is made available under aCC-BY 4.0 International license.

Figure 1-video 1 Coordination phenotypes of $ana1^{mecB}$, $ana1^{\Delta 1\text{-}762}$ and $ana1^{mecB}/ana1^{\Delta 1\text{-}762}$ flies. 6 flies from each genotype were transferred into vials without anaesthesia and their movements recorded. Flies were raised and tested at 25 ^{o}C.

Figure 2- video 1 Representative videos for each coordination phenotype. 10 flies from each genotype were transferred to a petri dish and recorded. Flies were raised and tested at 25 ^{o}C. No rescue (-), the flies couldn't stand; weak rescue (+), the flies could walk, but not climb; good rescue (++), the flies can could with difficulty; complete rescue (+++), wild type phenotype.

Figure 2- video 2 Climbing assays of indicated transgenic flies expressing Ana1 fragments in an $ana1^{null}$ background. Cohorts of 10 flies were scored for their ability to climb 5 cm in 30 s. Flies were raised and tested at 25 ^{o}C.

Figure 3-video 1 Movie shows Z stack volume of $ana1^{null}$ chordotonal organ transverse section revealing a missing basal body. Scale bar, 100 nm.

Figure 3-video 2 Movie shows Z stack volume of $ana1^{null}$ chordotonal organ transverse section revealing cilia and a basal body. Scale bar 500, nm.

Figure 1- figure supplement 1

bioRxiv preprint doi: https://doi.org/10.1101/2024.10.28.620588; this version posted October 31, 2024. The copyright holder for this preprint (which was not certified by peer review) is the author/funder, who has granted bioRxiv a license to display the preprint in perpetuity. It is made available under aCC-BY 4.0 International license.

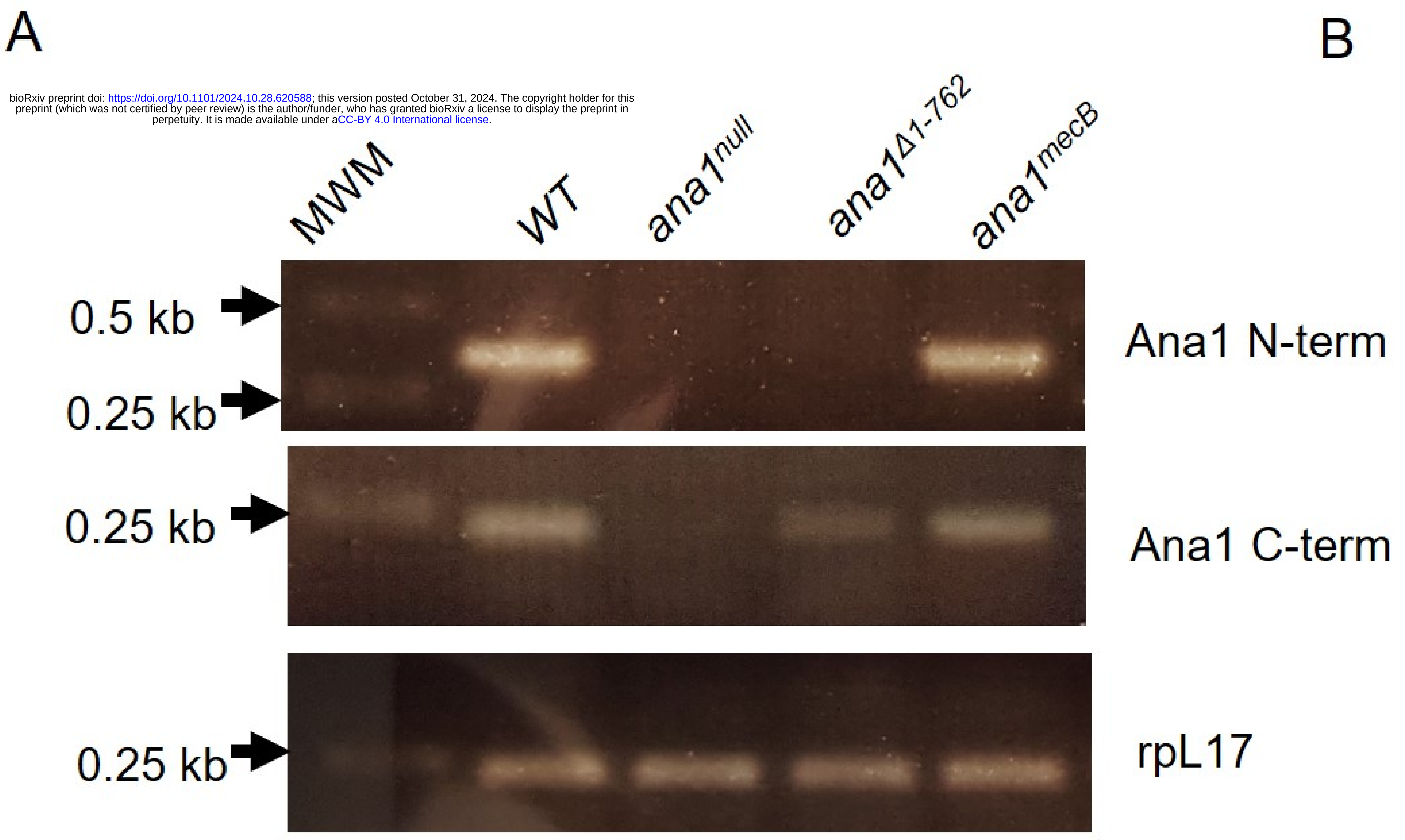

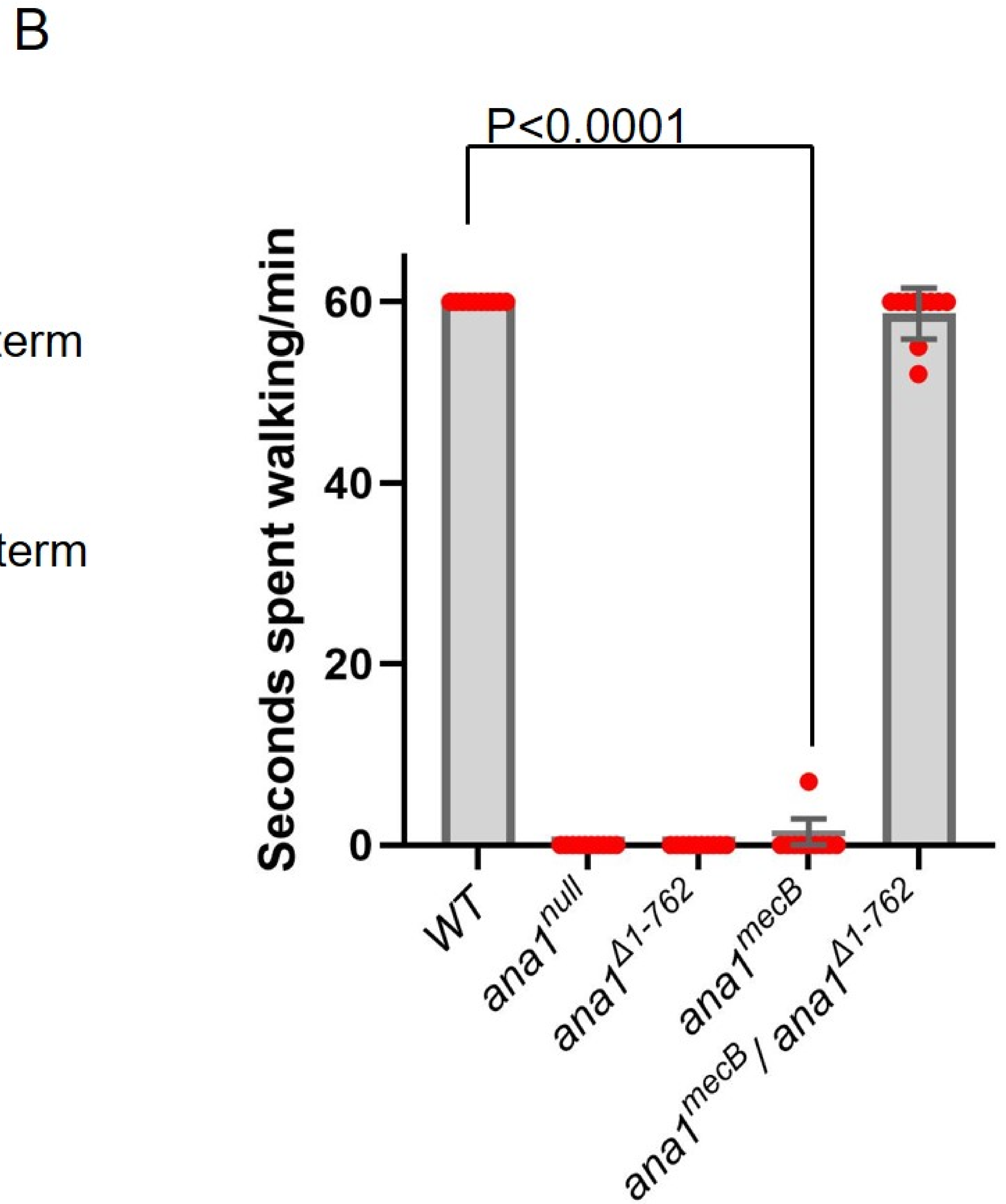

Figure 2- figure supplement 1

bioRxiv preprint doi: https://doi.org/10.1101/2024.10.28.620588; this version posted October 31, 2024. The copyright holder for this preprint (which was not certified by peer review) is the author/funder, who has granted bioRxiv a license to display the preprint in perpetuity. It is made available under aCC-BY 4.0 International license.

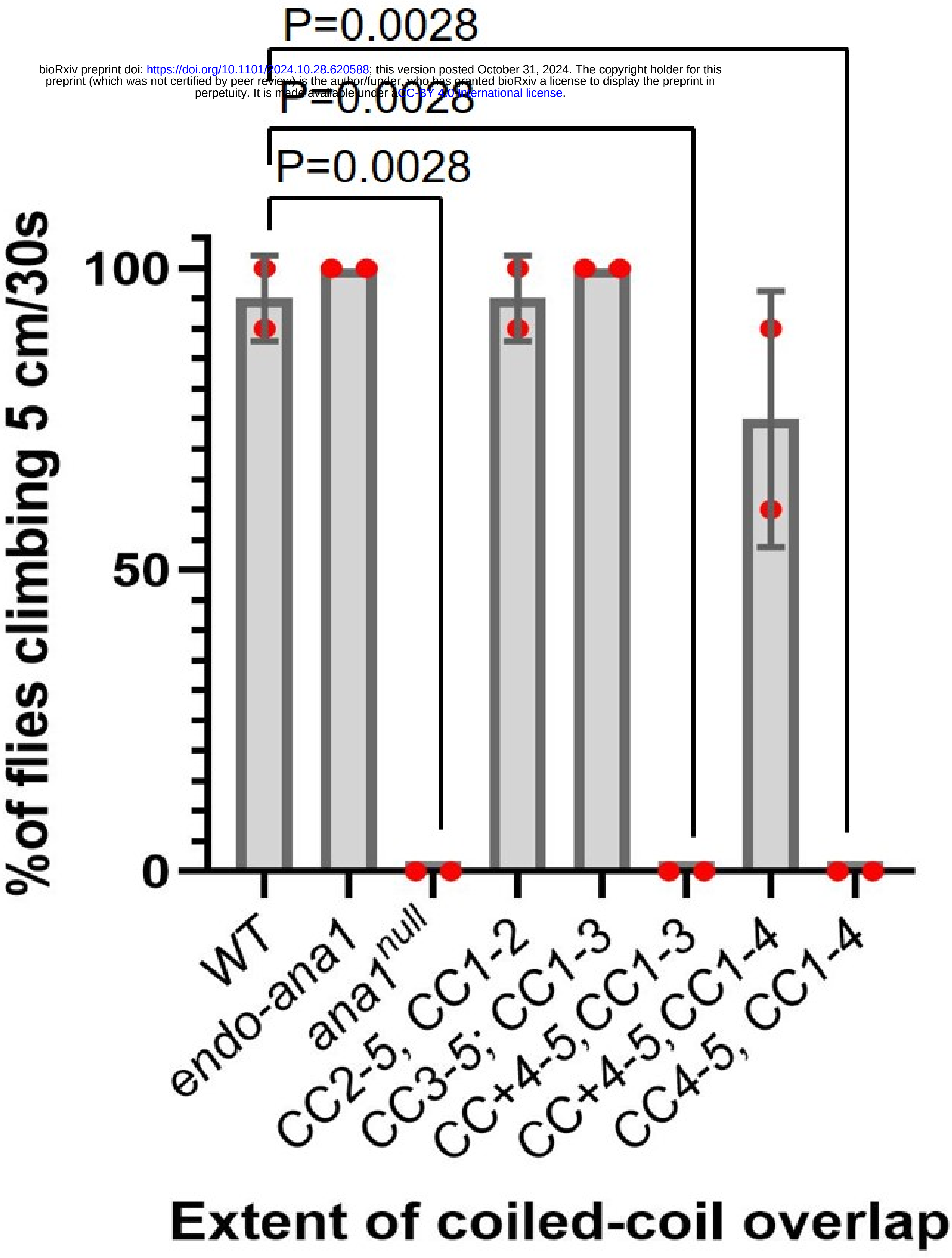

Figure 3- figure supplement 1

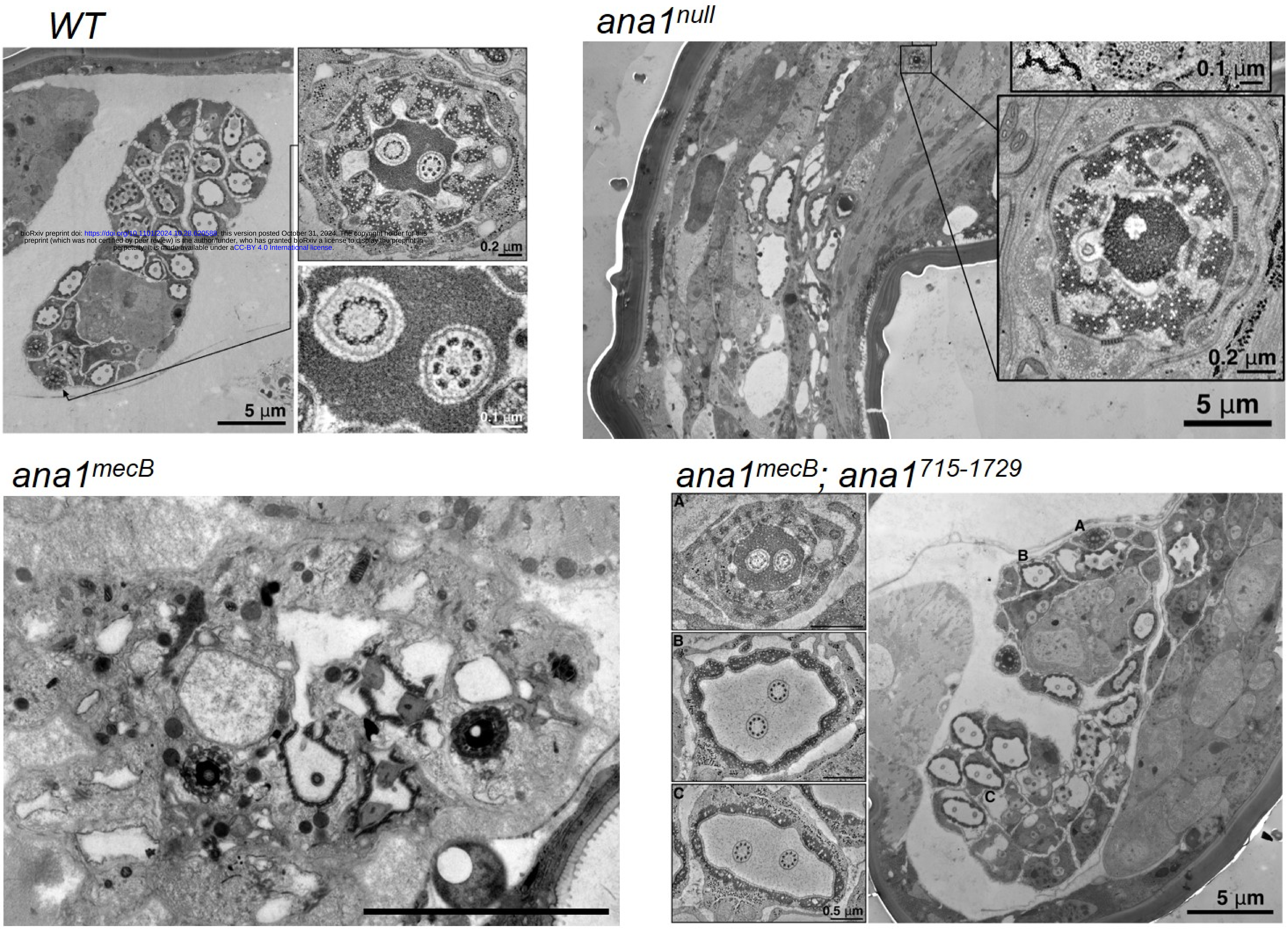

bioRxiv preprint doi: https://doi.org/10.1101/2024.10.28.620588; this version posted October 31, 2024. The copyright holder for this preprint (which was not certified by peer review) is the author/funder, who has granted bioRxiv a license to display the preprint in perpetuity. It is made available under aCC-BY 4.0 International license.

Figure 4- figure supplement 1

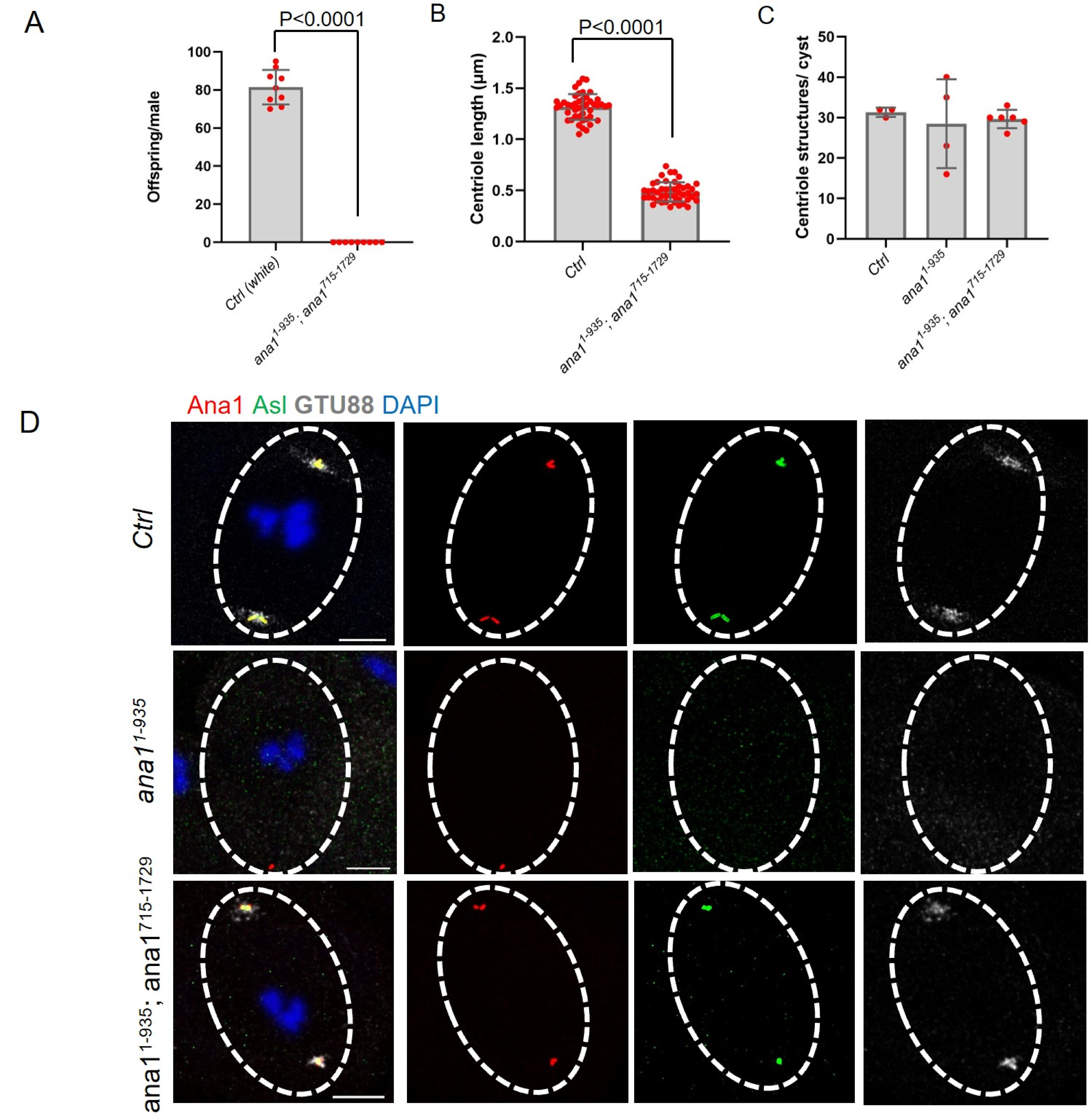

E

bioRxiv preprint doi: https://doi.org/10.1101/2024.10.28.620588; this version posted October 31, 2024. The copyright holder for this preprint (which was not certified by peer review) is the author/funder, who has granted bioRxiv a license to display the preprint in perpetuity. It is made available under aCC-BY 4.0 International license.

Centriole length

	endo-ana1$^{1-935}$ *CC1-3*	*endo-ana1$^{1-1120}$* *CC1-4*	*endo-ana1$^{1-1200}$* *CC1-5*	*endo-ana1$^{1-1430}$* *CC1-5+*	*endo-ana1*
ana1^{null}	no rescue 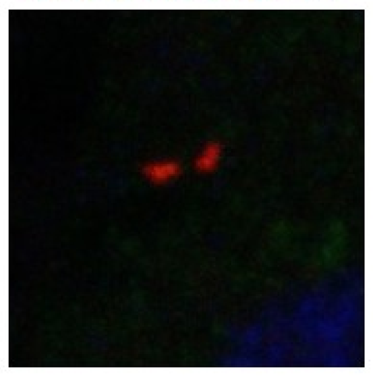	no rescue 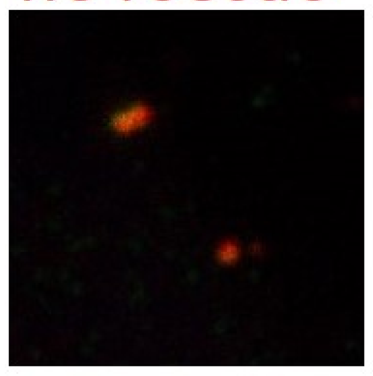	no rescue 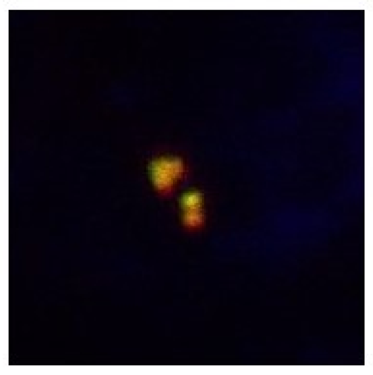	no rescue 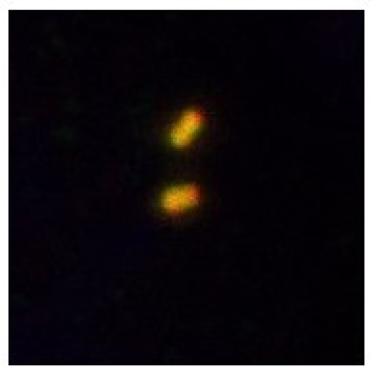	rescue 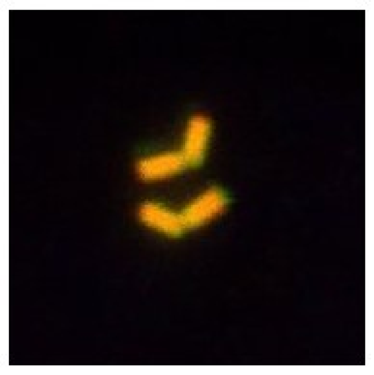
endo-ana1$^{715-1729}$ *CC3-5*	no rescue 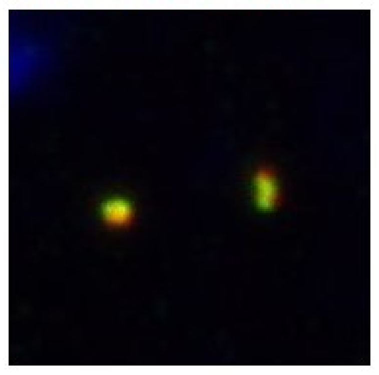	no rescue 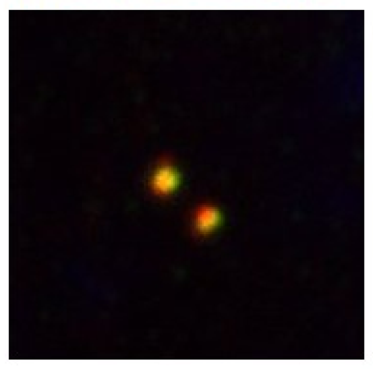	no rescue 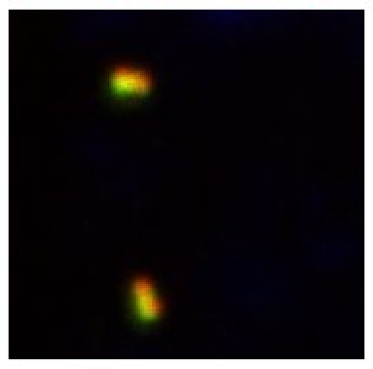	no rescue 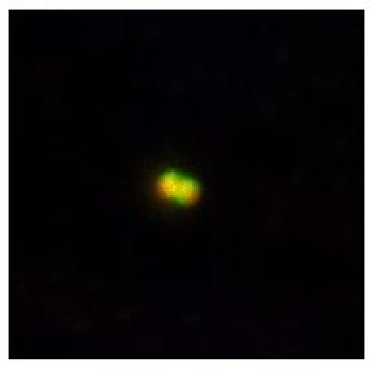	---

Figure 4- figure supplement 2

bioRxiv preprint doi: https://doi.org/10.1101/2024.10.28.620588; this version posted October 31, 2024. The copyright holder for this preprint (which was not certified by peer review) is the author/funder, who has granted bioRxiv a license to display the preprint in perpetuity. It is made available under aCC-BY 4.0 International license.

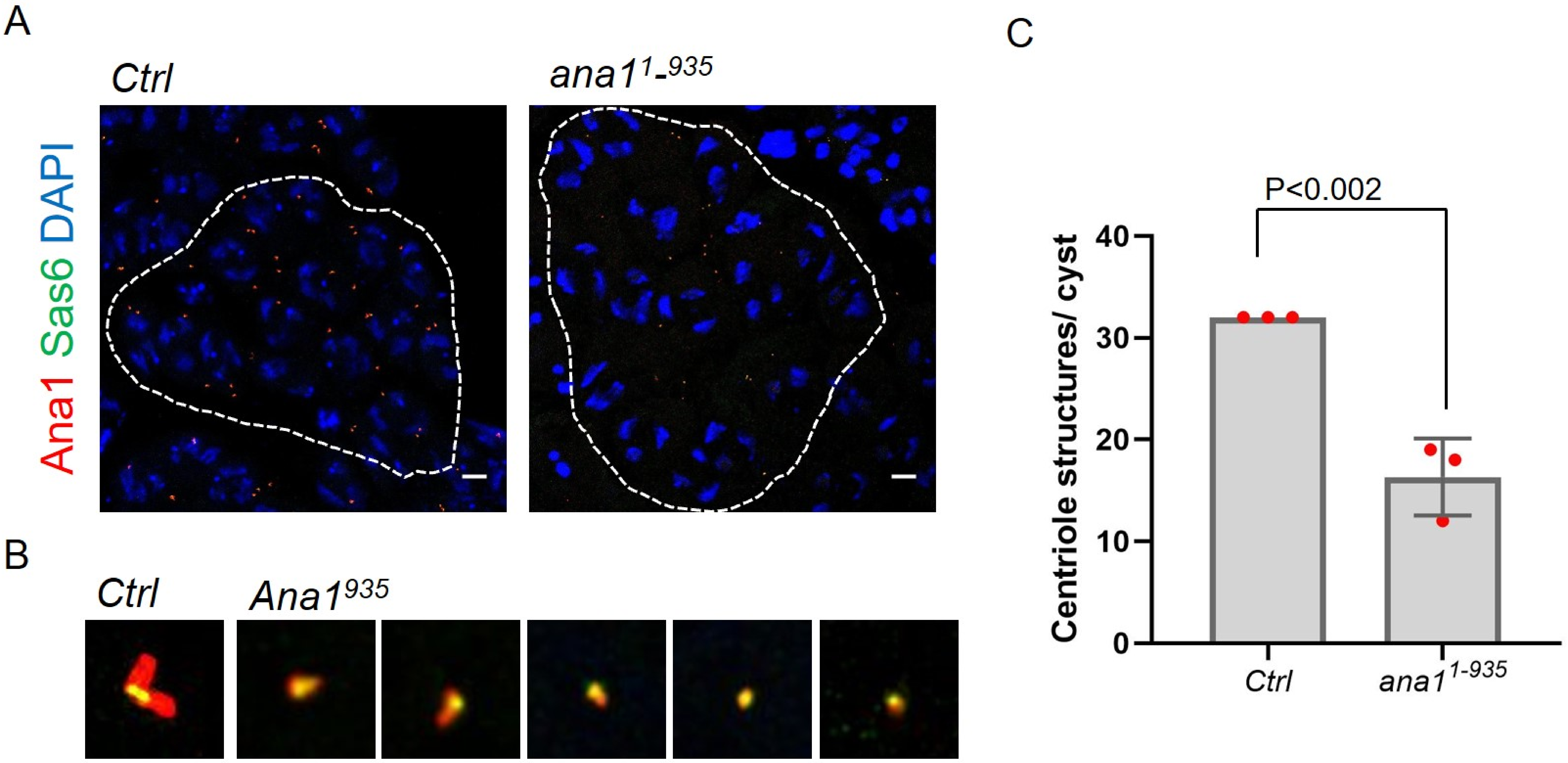

Supplement: 1 [file NIHPP2024.10.28.620588v1-supplement-1.pdf]
